# A retrospective study on the socio-demographic factors and clinical parameters of dengue disease and their effects on the clinical course and recovery of the patients in a tertiary care hospital of Bangladesh

**DOI:** 10.1371/journal.pntd.0010297

**Published:** 2022-04-04

**Authors:** Kazi Milenur Rahman Prattay, Md. Raihan Sarkar, Abu Zar Md. Shafiullah, Md. Saiful Islam, Sheikh Zahir Raihan, Nahid Sharmin

**Affiliations:** 1 Department of Pharmacy, University of Dhaka, Dhaka, Bangladesh; 2 Department of Pharmaceutical Technology, University of Dhaka, Dhaka, Bangladesh; 3 Department of Statistics, University of Dhaka, Dhaka, Bangladesh; 4 Department of Pharmaceutical Chemistry, University of Dhaka, Dhaka, Bangladesh; 5 Department of Clinical Pharmacy & Pharmacology, University of Dhaka, Dhaka, Bangladesh; RTI International, UNITED REPUBLIC OF TANZANIA

## Abstract

Dengue, a mosquito transmitted febrile viral disease, is a serious public health concern in Bangladesh. Despite significant number of incidences and reported deaths each year, there are inadequate number of studies relating the temporal trends of the clinical parameters as well as socio-demographic factors with the clinical course of the disease. Therefore, this study aims to associate the clinical parameters, demographic and behavioral factors of the dengue patients admitted in a tertiary care hospital in Dhaka, Bangladesh during the 2019 outbreak of dengue with the clinical course of the disease. Data were collected from the 336 confirmed dengue in-patients and analyzed using SPSS 26.0 software. Majority of the patients were male (2.2 times higher than female) who required longer time to recover compared to females (p < 0.01), urban resident (54.35%) and belonged to the age group of 18–40 years (73.33%). Dengue fever (90.77%) and dengue hemorrhagic fever (5.95%) were reported in most of the dengue patients while fever (98%) was the most frequently observed symptom. A significantly positive association was found between patient’s age and number of manifested symptoms (p = 0.013). Average duration of stay in the hospital was 4.9 days (SD = 1.652) and patient’s recovery time was positively correlated with delayed hospitalization (p < 0.01). Additionally, recovery time was negatively correlated with initial blood pressure (both systolic (p = 0.001, and diastolic (p = 0.023)) and platelet count (p = 0.003) of the patients recorded on the first day of hospitalization. Finally, a statistical model was developed which predicted that, hospital stay could be positively associated with an increasing trend of temperature, systolic blood pressure and reduced platelets count. Findings of this study may be beneficial to better understand the clinical course of the disease, identify the potential risk factors and ensure improved patient management during future dengue outbreaks.

## Author summary

Dengue, a mosquito transmitted febrile viral disease, is a serious public health concern in Bangladesh. Despite soaring occurrence of the disease and associated deaths reported over the years, there are not adequate studies demonstrating the effect of the socio-demographic and clinical parameters on the clinical course of the disease. Hence, the objective of this study is to correlate these factors with the disease severity and recovery of the in-patients during the 2019 dengue outbreak in Bangladesh. We reached the medical records of 336 confirmed hospitalized dengue patients of a tertiary care hospital in Dhaka city and gathered their socio-demographic and clinical information. Majority of the dengue patients were male, urban resident, 18–40 years of age and the number of symptoms had a positive correlation with age. Patients with lower platelet count and blood pressure stayed longer in the hospital. Male patients as well as patients with delayed hospitalization also had a longer recovery period. Finally, we constructed a statistical model that predicted that hospital stay could be positively associated with an increasing trend of temperature, systolic blood pressure and reduced platelets count. Information from this study may be beneficial to better understand the clinical course of dengue, identify the potential risk factors and ensure improved patient management during future dengue outbreaks.

## Introduction

Dengue is a systemic, febrile disease caused by a mosquito-borne virus known as Dengue virus (DENV) and is one of the major public health problems in tropical and subtropical countries [1,2]. DENV is a small (50nm) enveloped virus that belongs to the genus Flavivirus from family “Flaviviridae” [3]. Infection by any of the DENV serotype provides lifelong immunity against that particular serotype but only a few months of immunity against others. Rather multiple infections with different serotypes or, secondary infection with another serotype increases the chance of severe clinical manifestations of dengue [4].

Over the last two decades, the incidence of this fastest-spreading infectious disease has increased more than eight-fold, owing to increased regional expansion into new countries and more recently, from urban to rural settings [5–7]. South-East Asian countries are estimated with about 75% of the total disease cases and economic burden caused by dengue fever worldwide [8]. Dengue fever has been the leading causes of hospitalization and death in children and adults of this region in the last 10–15 years [4].

Although majority of the patients with dengue infection remains asymptomatic, about 25% of the infected patients might suffer from a self-limiting febrile syndrome along with mild to moderate hematological and other complications after an incubation period of 4 to 7 days (range 3–14 days). A small proportion of patients suffer from severe complications as well [4,9]. A new single parameter classification system has been introduced by WHO in 2009 for symptomatic dengue infections but it often does not fit the poor healthcare facility of the endemic regions since it requires a high level of nursing and medical interventions [10,11]. According to Bangladesh’s national guideline for clinical management of dengue syndrome, there are four different classes of dengue illness, namely- undifferentiated dengue fever (UDF), dengue fever (DF), dengue hemorrhagic fever (DHF) and expanded dengue syndrome (EDS) [4]. Although these four classes are not entirely based on severity but very often UDF is a fairly mild form of the dengue (indistinguishable from other viral infections), DF is mild to moderate, DHF is moderate to severe form (specially, in case of dengue shock syndrome (DSS) and EDS is pretty severe which is associated with comorbidities, coinfections and often leads to organ failure [4].

Bangladesh experienced her first largest dengue outburst in 2002 (until 2018) with 6132 reported cases alongside 56 confirmed deaths after the first official outbreak in 2000 [12,13]. A five-year (2013–2017) study displayed that complications from DHF/DSS were the major causes of death (65%) of dengue patients in Bangladesh and >70% of the deaths occurs within 24 hours of being admitted to the hospital [14]. Several studies have been performed to understand dengue epidemiology and clinical presentations in Bangladesh [15–17]. But recently, changing clinical patterns and unusual manifestations of dengue fever were reported [14,18–20]. Dengue endemic in 2018 came with a special pattern of clinical observations which include less rash, more leukopenia, early positive Tourniquet test, diarrhea, high AST, ascites etc [19]. Febrile diarrhea, ascites, abdomen pain, pleural effusion, edema in gall bladder, hepatomegaly were more frequently reported in 2019 outbreak than before [18]. Although delayed hospitalization has been found as a risk factor of DHF, there is a lack of sufficient analytical data regarding the mutual relationship among delayed hospitalization, duration of febrile phase, length of hospitalization and severity of dengue fever [21]. Information from some investigations suggest daily platelet and/or hematocrit count (Hct), degree of thrombocytopenia to be predictive as well as recovery parameter of DHF/DSS patients [22,23]. In addition, patients with severe dengue/DHF exhibit significant differences in the temporal trend of their systolic blood pressure compared to other patients [24]. Nonetheless, the number of studies conducted involving analyses on daily platelet count, Hct count and blood pressure of dengue patients are scarce worldwide. Hence, it is crucially important to analyze the clinical manifestations of dengue endemic in recent years, co-relate the disease severity with this changing patterns to develop and modify an optimum guideline for the early and appropriate detection and management of the disease which in turn may significantly reduce the sufferings of the patients and lessen the economic burden of the nation thereby.

The present study demonstrates the conventional and unusual signs and symptoms of dengue fever outbreak in 2019 in a tertiary care teaching hospital of Bangladesh. The objective of this survey is to investigate and relate the severity of the disease with different potential socio-demographic and clinical parameters such as age, sex, blood pressure, fever, platelet count, duration of febrile phase and delay in hospitalization. In addition, this work also aims to develop a predictive statistical model which, depending on the temporal change of the above mentioned factors will correlate the recovery time/hospital stay of the dengue patients. To the best of our knowledge this is the first report in Bangladesh showing how the temporal trend of socio-demographic and physiologic parameters changed in dengue patients and how these factors might have affected the clinical course and/or complications of dengue illness in 2019 which, in turn, may aid to devise more optimized diagnosis and management of future dengue patients.

## Methodology

### Ethics statement

This retrospective study was conducted following the principles of the World Medical Association (WMA) Declaration of Helsinki. The Department of Pharmacy Academic Committee, University of Dhaka reviewed and approved the protocol of the survey (approval number 311). Approval to conduct the study was also given by the authority of Dhaka Medical College Hospital, Dhaka, Bangladesh, the data set owner of this study (approval ID: 12684). The survey collected data only from the records of dengue patients admitted into this hospital from July to August 2019 and anonymity of the patients was strictly maintained.

### Study design, setting and participants

This study was designed as a uni-centered, retrospective study with an aim to gather information associated with dengue fever at individual level. It was conducted to get a broader view of perception on different socio-economic indicators and other potential physiological and non-physiological parameters that might affect the health condition and days of hospitalization of the patients. Current study deals with fraction of patients who were admitted in the temporary dengue ward in one of the topmost tertiary government hospitals of Bangladesh, located in the capital city Dhaka from July to August 2019.

A total of 336 patients data were collected for this study and all of them fulfilled the following two criteria of inclusion: 1. at least once found positive in NS1 antigen test or, IgM/IgG antibody ratio test in the hospital 2. minimum one night of hospitalization. Data were collected using a self-made proforma case record form from the medical records kept by the hospital after releasing the patients. Proper procedures were followed to receive permission from Department of Pharmacy, University of Dhaka and the hospital authority to conduct this study (Reference no. of the application: 12684).

Data like demography, sex, age, days of hospitalization, symptoms, duration of fever, type of dengue fever were recorded. The concerned hospital authority classified the dengue patients into four different types (UDF, DF, DHF and EDS) according to Bangladesh’s national guideline for clinical management of dengue syndrome, and the data was documented accordingly [4]. Clinical parameters like average systolic and diastolic blood pressure, temperature and platelet count of the patients were recorded as well on a day-to-day basis during their residence in the hospital. All the collected data were organized by using Microsoft Excel (2015) software.

### Statistical analysis

Different continuous variables were analyzed for descriptive statistics like mean and standard deviation. On the other hand, various categorical variables were evaluated for descriptive statistics like frequency distribution along with percentage and valid percentage. In all appropriate cases, 95% confidence interval was calculated. For testing equality of means for two categories of categorical variables (for example: sex, area), the independent sample T-test was performed whereas for categorical variables with more than two categories (like age group, type of fever, etc.), the corresponding hypotheses were tested using ANOVA (Analysis of Variance) F test. Post Hoc test was performed using Least Significant Difference (LSD) method to determine the true source of differences only when the ANOVA test showed statistically significant result (p < 0.05). For two categorical variables, the Chi-square test was used to explore any association between them. The relationship among two numerical variables is further studied using Pearson’s correlation coefficient. Finally, a linear model was constructed using a conditional model (mixed-effects model) to demonstrate the association of recovery time with the temporal trends of temperature, blood pressure and platelet count in the study subjects. All the statistical analyses were performed by using IBM SPSS Statistics (for windows), version 26.0.

## Results

### Association of the socio-demographic characteristics of the patients with the precipitation of dengue

Of the total studied population, age and residential area related data were available for 315 and 276 dengue patients respectively while gender information was available for all the 336 patients (S1 Table). Majority of the cases (73.33%) were found between the ages of 18 and 40. Age groups of 41–60 years and <18 years were presented with 15.24% and 9.52% of cases respectively while the least percentage of cases (1.90%) were exhibited by patients >60 years of age (S1 Table). Although the gender distribution across the four age groups was statistically the same (p > 0.05), majority (68.75%) of the total dengue cases were found to be male. Additionally, it was found that people of urban area (54.35%) were infected more than the rural region of the country (S1 Table).

### Distribution of different classes of dengue cases among study population in terms of age and sex

**Fig 1:**
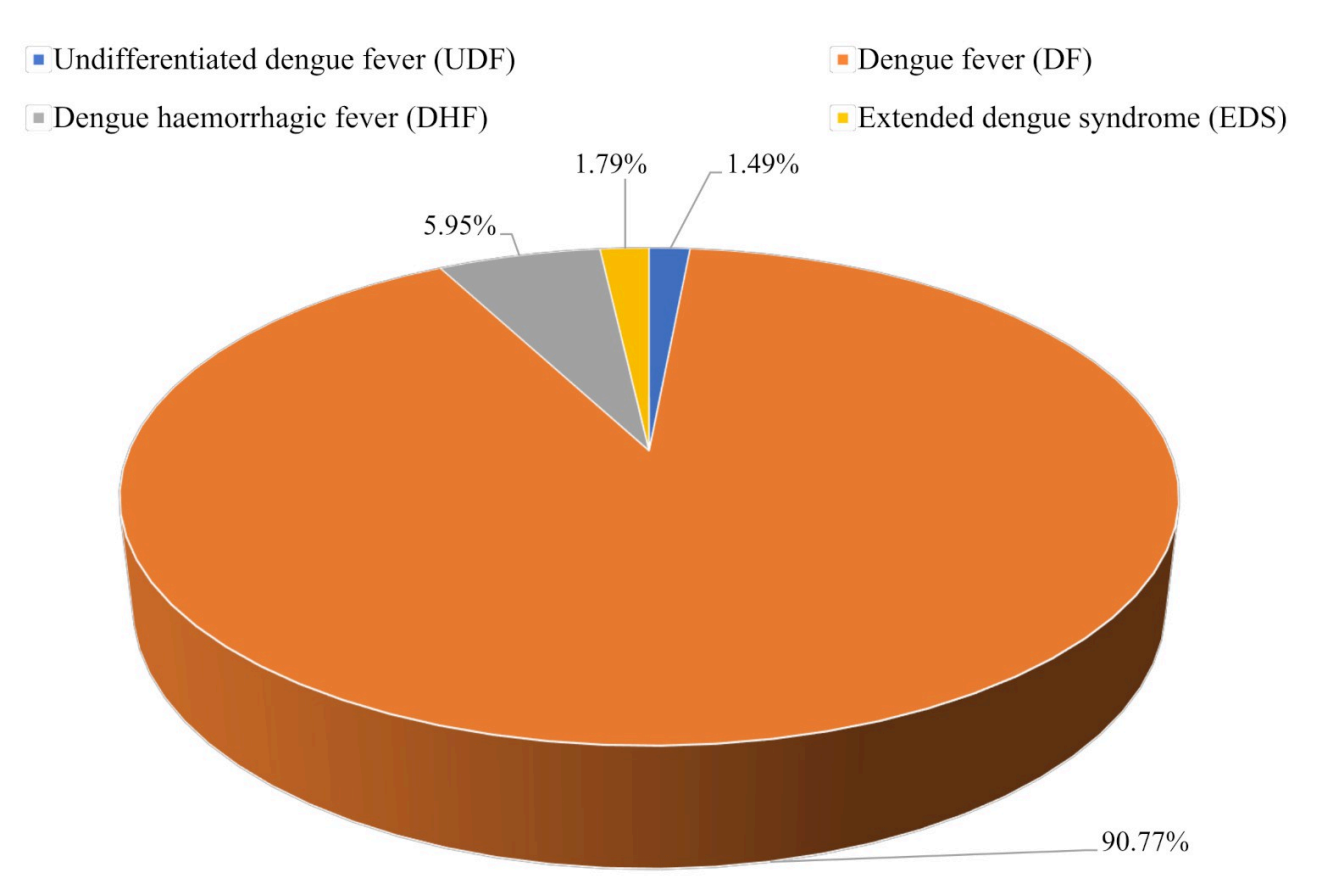
Distribution of dengue cases by severity among study population.

Among a total of 336 patients, 305 (90.77%) of them suffered from DF which was much greater compared to other classes of dengue illness (Fig 1). While DHF was identified in 20 (5.95%) patients, expanded dengue syndrome (EDS) and undifferentiated dengue fever (UDF) were confirmed only in six (1.79%) and five (1.49%) patients respectively (Fig 1). It is evident from Fig 1 that the majority of the dengue patients admitted into that specific hospital suffered from mild to moderate form of the disease followed by the more severe DHF type of dengue. Only a small portion of the patients demonstrated severe dengue associated with comorbidities/coinfections (Fig 1).

Present study also showed that, DF was identified by all age groups of patients (S1 Fig). Most of the patients of <18 and 41–60 years of age exhibited DF (93.3% and 97.9% respectively) and a few were detected with DHF (6.7% and 2.1% respectively) (S1 Fig). Patients falling in the age group of 18–40 years showed all forms of the disease with DF as the predominant type (87.9%) (S1 Fig). It was noteworthy that the elder age group (>60 years) was suffering from DF only (100%) (S1 Fig).

Type of dengue fever was found to be significantly associated with sex (p<0.05) (S2 Fig). It was interesting to notice the number of female patients were considerably lower for all types of dengue (5% for DHF, 33.1% for DF). While no female patients were detected with UDF, EDS was equally exhibited by both gender groups (S2 Fig).

### Clinical manifestations displayed by the patients and total number of symptoms exhibited by different age groups

The current study indicated that on an average 2.68 (95%CI: 2.51–2.84) numbers of clinical symptoms were manifested in the study population where fever (98%) had been witnessed as the most common clinical sign (S2 Table). Body ache (39.28%), vomiting (29.5%), headache (21.1%), anorexia (17.9%), abdominal pain (12.2%) were some of the other most frequently observed clinical features (S3A Fig and S2 Table).

Patients older than 60 years of age were found to manifest a maximum of 3.33 number of symptoms on average (95%CI: 1.27–5.40) (S3A Fig and S3 Table). However, the average number of features displayed decreased in the patients of age groups 41–60 and 18–40 to 3.23 (95%CI: 2.73–3.73) and 2.56 (95%CI: 2.37–2.75) respectively (S3A Fig and S3 Table). Interestingly, this parameter was found to be the minimum (2.23; 95%CI: 1.78–2.69) in patients below 18 years of age (S3A Fig and S3 Table).

Next, we wanted to see if the total number of symptoms across the age groups were significantly different from each other by using one-way ANOVA and Post Hoc multiple comparison tests as well. A significant correlation was found between the number of clinical symptoms displayed between the patients and their age (p = 0.013), F (3, 311) = 3.675, p = 0.013. Pairwise comparisons of the means using LSD revealed that mean total number of symptoms in patients of 41–60 years was significantly higher (at 0.05 level) from that of patients less than 18 years of age and 18–40 age group (S3B Fig and S3 Table). This finding may be of importance while suspecting dengue cases over different age groups even before diagnosis. However, no significant relationship was found between sex and total number of symptoms displayed by a patient in any age groups (p > 0.05) (S3C Fig).

### Temporal trends of dengue patient’s clinical parameters and relation of these parameters with recovery time

#### A. Correlation of patients’ recovery time with blood pressure (BP) and changing pattern of the parameter during hospital stay

Lower blood pressure has been associated with dengue cases [24] and hence both systolic blood pressure (SBP) and diastolic blood pressure (DBP) of the dengue patients had been observed and analyzed for the first six days of hospitalization to find out any correlation between BP and hospital stay.

**Fig 2:**
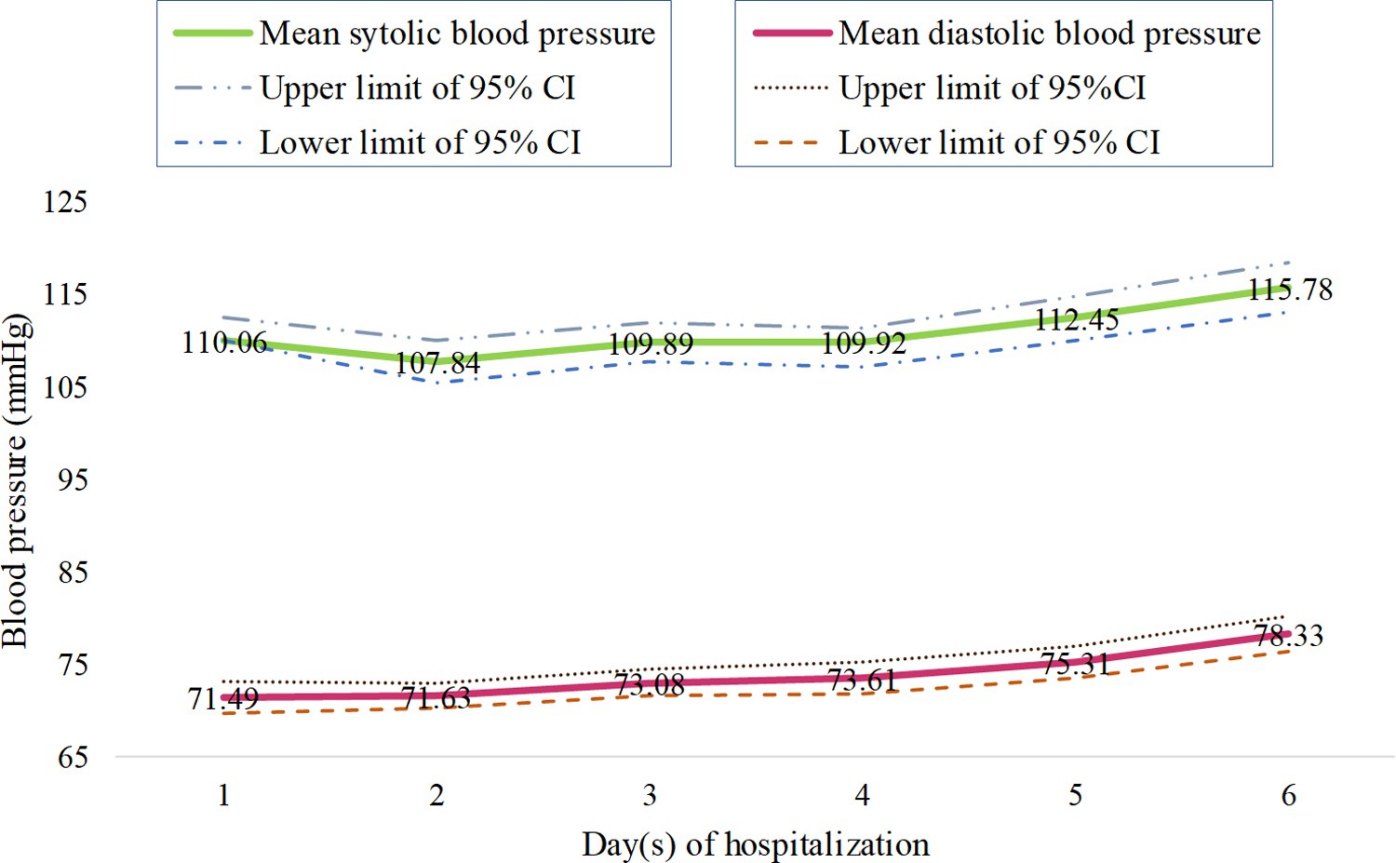
Mean blood pressure of patients (both systolic and diastolic) at the consecutive day(s) of hospitalization.

On the day of hospitalization, a mean systolic and diastolic blood pressure of 110.06 mmHg (95%CI: 107.50 to 112.61) and 71.49 mmHg (95%CI: 73.16 to 69.81) respectively were recorded for dengue patients both of which were significantly (p< 0.05) lower than the normal blood pressure value (120/80 mmHg) (Fig 2). This study showed that duration of stay in the hospital was significantly negatively correlated with the initial systolic and diastolic blood pressure of the patients on the day of hospitalization at 0.001 and 0.023 level respectively (Fig 2).

The mean systolic blood pressure decreased on the second day of hospitalization to 107.84 mmHg (95%CI: 105.54 to 110.15) and then started to go up and reached a maximum average value of 115.78 mmHg (95%CI: 113.18 to 118.37) on the sixth day of hospitalization (Fig 2). On the other hand, mean diastolic blood pressure rose gradually from the first day of admission from 71.49 mmHg (95%CI: 73.16 to 69.81) and continued to do so upto the sixth day 78.33 mmHg (95%CI: 76.38 to 80.29) of hospital stay (Fig 2).

**Fig 3:**
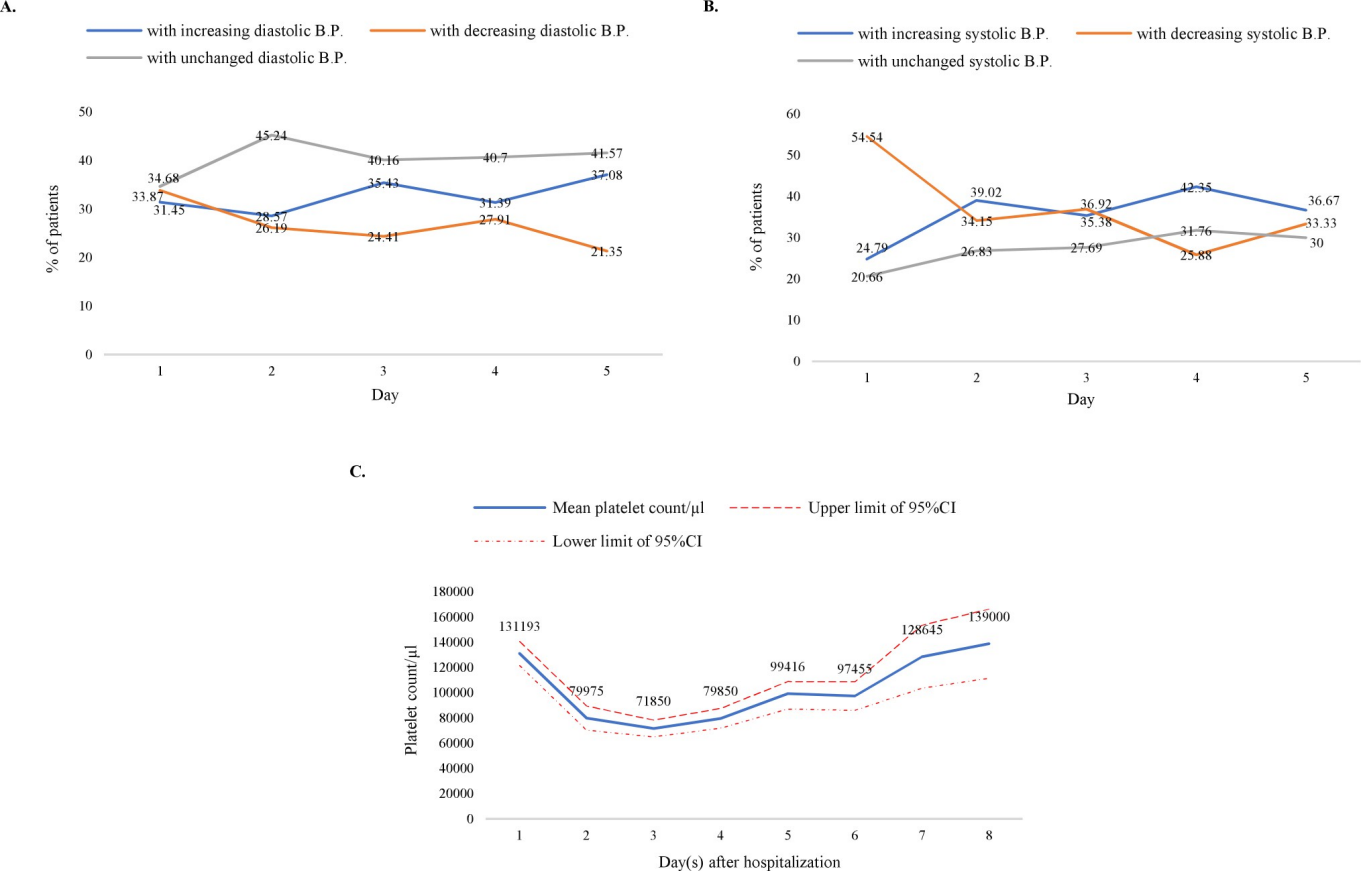
Temporal trends of dengue patient’s clinical parameters and correlation of these parameters with recovery time. (A) Changing tendency of diastolic blood pressure after day(s) of hospitalization. (B) Changing tendency of systolic blood pressure after day(s) of hospitalization. (C) Mean platelet count of patients versus days after hospitalization.

Systolic blood pressure was found to fall in a higher percentage of patients (54.54%) when compared to diastolic blood pressure (33.87%) after the first day of hospitalization. The decrease of diastolic blood pressure was observed in 33.87%, 26.19%, 24.41%, 27.91 and 21.35% of patients from day 1 to day 5 respectively (Fig 3A). However, 55.54%, 34.15%, 36.92%, 25.88%, 33.33% of the patients experienced a lowering in their systolic blood pressure going from 1 to 5 days of their hospitalization (Fig 3B) suggesting the systolic blood pressure in dengue patients is more prone to change than the diastolic pressure.

#### B. Temporal trend of platelet counts and patients’ recovery time

Platelet count data was available for at least two days for 269 patients. It was reported that, platelet counts have a correlation with the severity of the DHF/DSS [23]. Hence the mean platelet count of all the patients was analyzed on a day to day basis. It was found that platelet count decreased during the first 3 days of hospitalization with a lowest mean platelet count of 71,850 on the 3^rd^ day. After that, a rise in platelet count was observed from day 4 (99,416) and it reached the peak value on 8^th^ day (1,39,000) except for a slight decrease on day 6^th^ (97,455). Platelet count on day 5 and day 7 were 99416 and 97455 respectively (Fig 3C).

It was also observed that, of the study population, 31.97% of the patients experienced no thrombocytopenia (platelet count < 1,00,000) at any stage of his/her stay in the hospital. A maximum of 33.09% study patients suffered from moderate thrombocytopenia (platelet count: 20,000 to 50,000) and minimum of 7.06% suffered from severe thrombocytopenia (platelet count < 20,000) (S4 Fig). Around 28% of the patients suffered from mild (platelet count: more than 50,000 to 100,000) thrombocytopenia (S4 Fig and Table 1). However, no significant association was found among patients’ age and severity and/or presence of thrombocytopenia (at 0.05 level) (Table 1).

**Table 1:** Age wise distribution of severity of thrombocytopenia.

Age	Minimum Platelet Count				
	< 20000 (severe thrombocytopenia)	20,000–50,000 (moderate thrombocytopenia)	50,001–1,00,000 (mild thrombo-cytopenia)	>1,00,000 (no thrombocytopenia)	Total
**<18**	1	5	3	9	18
	5.55%	27.78%	16.67%	50%	100%
**18–40**	9	75	63	57	204
	4.41%	36.76%	30.88%	27.94%	100%
**41–60**	6	6	9	20	41
	14.63%	14.63%	21.96%	48.78%	100%
**>60**	3	3	0	0	6
	50%	50%	0%	0%	100%
**Total**	19	89	75	86	269
	7.06%	33.09%	27.88%	31.97%	100%

Among the study population, platelet count data on the first day of hospitalization was available for 132 patients. Of them, only 29.54% of the patients were admitted with thrombocytopenia (platelet count < 1,00,000) and none of them had severe thrombocytopenia (platelet count < 20,000) on the day of admission. Nearly 23% and 7% of all the patients exhibited mild (platelet count: more than 50,000 to 1 lakh) and moderate (platelet count: 20,000 to 50,000) thrombocytopenia respectively on their first day in the hospital. It is evident from Table 2 that, a significantly negative correlation (p = 0.003) exists between initial platelet count and duration of stay in the hospital (Table 2).

**Table 2:** Distribution of cases according to the recovery time and platelet count on the day of admission (data available for 132 patients).

Platelet count at admission		Duration of stay in the hospital (recovery time)								Total
		1	2	3	4	5	6	7	8	
< 20000 (Severe thrombocytopenia)	Count	0	0	0	0	0	0	0	0	0
	% of patients	NA	NA	NA	NA	NA	NA	NA	NA	NA
20,000–50,000 (Moderate thrombocytopenia)	Count	0	0	0	6	3	0	0	0	9
	% of patients	0.0%	0.0%	0.0%	66.7%	33.3%	0.0%	0.0%	0.0%	100.0%
50,001–1,00,000 (Mild thrombocytopenia)	Count	0	0	6	9	6	6	3	0	30
	% of patients	0.0%	0.0%	20.0%	30.0%	20.0%	20.0%	10.0%	0.0%	100.0%
>1,00,000 (Thrombocytopenia absent)	Count	0	6	6	54	12	3	9	3	93
	% of patients	0.0%	6.5%	6.5%	58.1%	12.9%	3.2%	9.7%	3.2%	100.0%

NA: not applicable.

### Association between the delay in hospitalization with patients’ average recovery time and habitat

**Fig 4:**
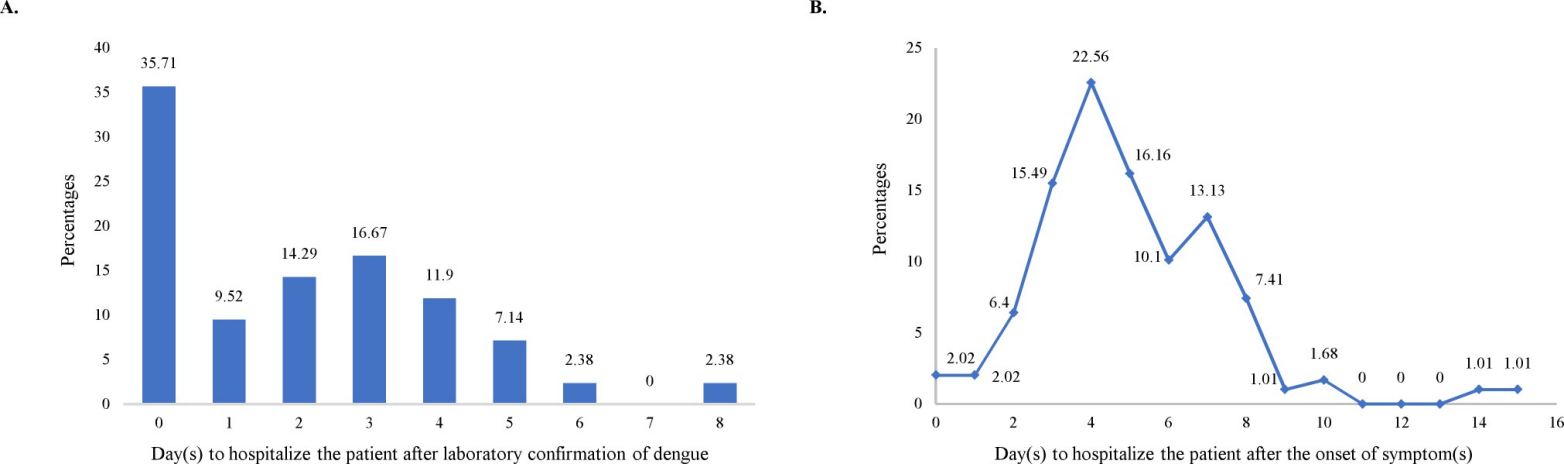
Association of the average recovery time of the patients with delay in the hospitalization. (A) Distribution of patients based on their delay in hospitalization after the confirmation of DENV infection, (B) Distribution of patients based on their delay in hospitalization after the onset of symptoms.

Patients were admitted into the hospital within 0 (same day of diagnosis) to 7 days (2.1 days in average; SD = 2.019) after confirmation of DENV infection by serological test(s). In most of the cases the admission was on the same day of being seropositive or on the third day (35.71% and 16.67% of patients respectively) (Fig 4A). Patients’s recovery time was found to be significantly positively correlated (Pearson’s correlation: 0.296) with this delayed hospitalization after serological confirmation of dengue (p<0.05).

There was a time lapse of 5 days on average (SD = 2.44, range: 0 to 15 days) between the first onset of symptoms (mostly fever) and hospitalization of the patients. In most cases (22.56%), patient got hospitalized after 4 days of onset of fever (Fig 4B). Patients’ recovery time was found to be significantly positively correlated (Pearson’s correlation: 0.323) to the delay in hospitalization after the onset of fever (at 0.01 level).

One-way ANOVA test also indicated a significant difference in the delay of hospitalization after onset of symptoms among the different types of dengue patients, F (3, 293) = 4.774, p = 0.003. Pairwise comparisons of the means using LSD revealed that average delay in hospitalization after the onset of symptoms in UDF patients was significantly lower (at 0.05 level) than that of patients suffering from any other types of dengue fever (Table 3).

**Table 3:** Delay in hospitalization after the onset of symptoms and the type of dengue fever.

Type of fever	UDF	DF	DHF	EDS
Delay in hospitalization after the onset of symptoms (day(s))	0.00	5.03	5.50	4.33
95% Confidence interval (CI)	0.00–0.00	4.74–5.32	4.42–6.58	2.17–6.50
Std. error	0.00	0.148	0.513	0.843

^a,b^ Fever types with similar superscripts significantly (at 0.05 level) differ from each other in terms of average number of days lapsed in between the onset of symptoms and hospitalization of the patient.

**Fig 5:**
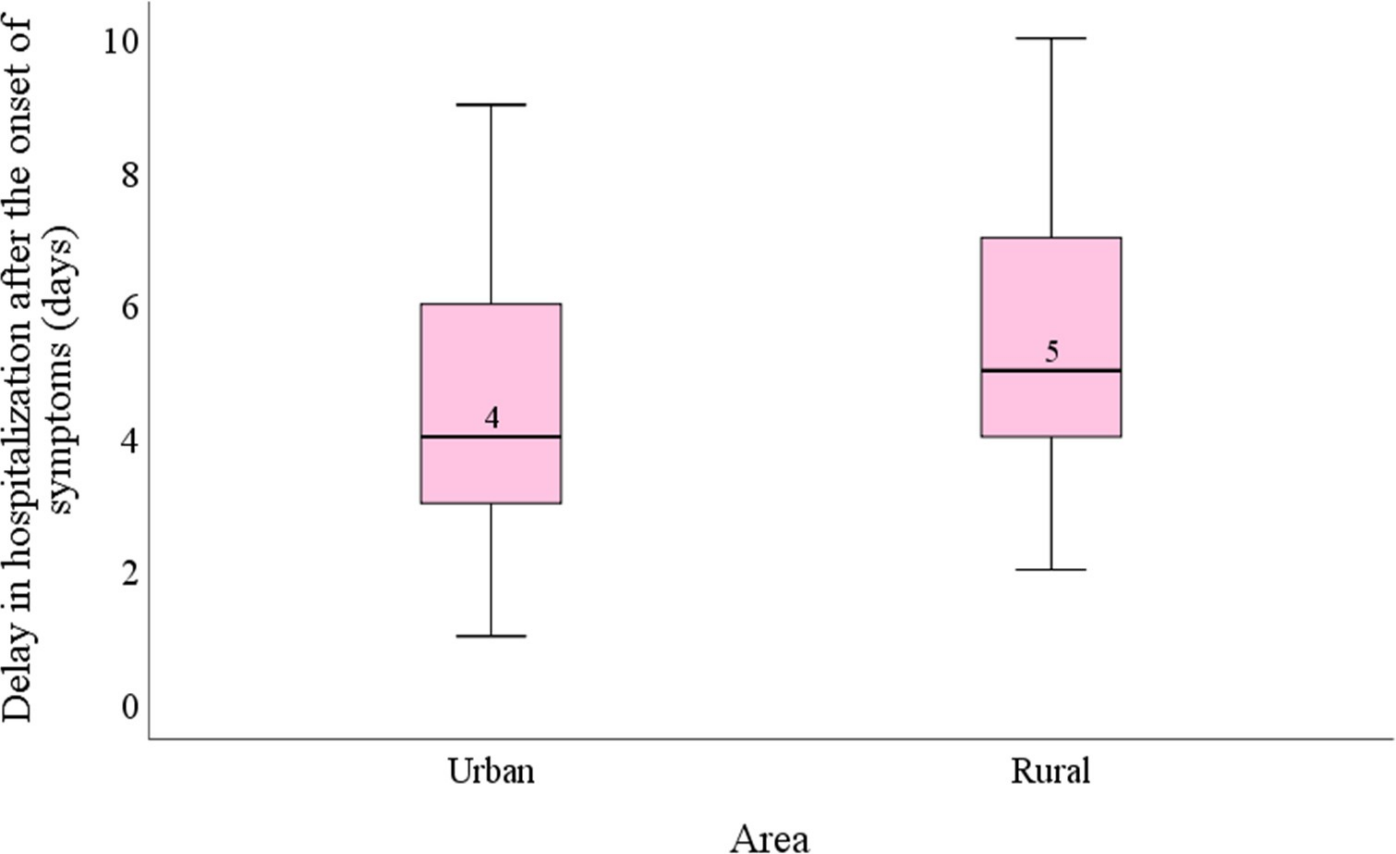
Relationship between the area of residence of the patients and delay in hospitalization after the onset of symptoms.

Delay in hospitalization after the onset of symptoms was found to be significantly associated with the habitat of the patients (p < 0.05). Patients from the urban area were hospitalized after an average of 4.5 (95%CI: 4.22–4.82) days of onset of first symptom which is lower than that (5 days; 95%CI: 4.57–5.45) of patients from rural areas of the country (Fig 5) suggesting there may be a lack of awareness and/or understanding about the disease in the countryside.

### Correlation of various demographic factors and type of dengue fever with the length of hospital stay of the patients

**Fig 6:**
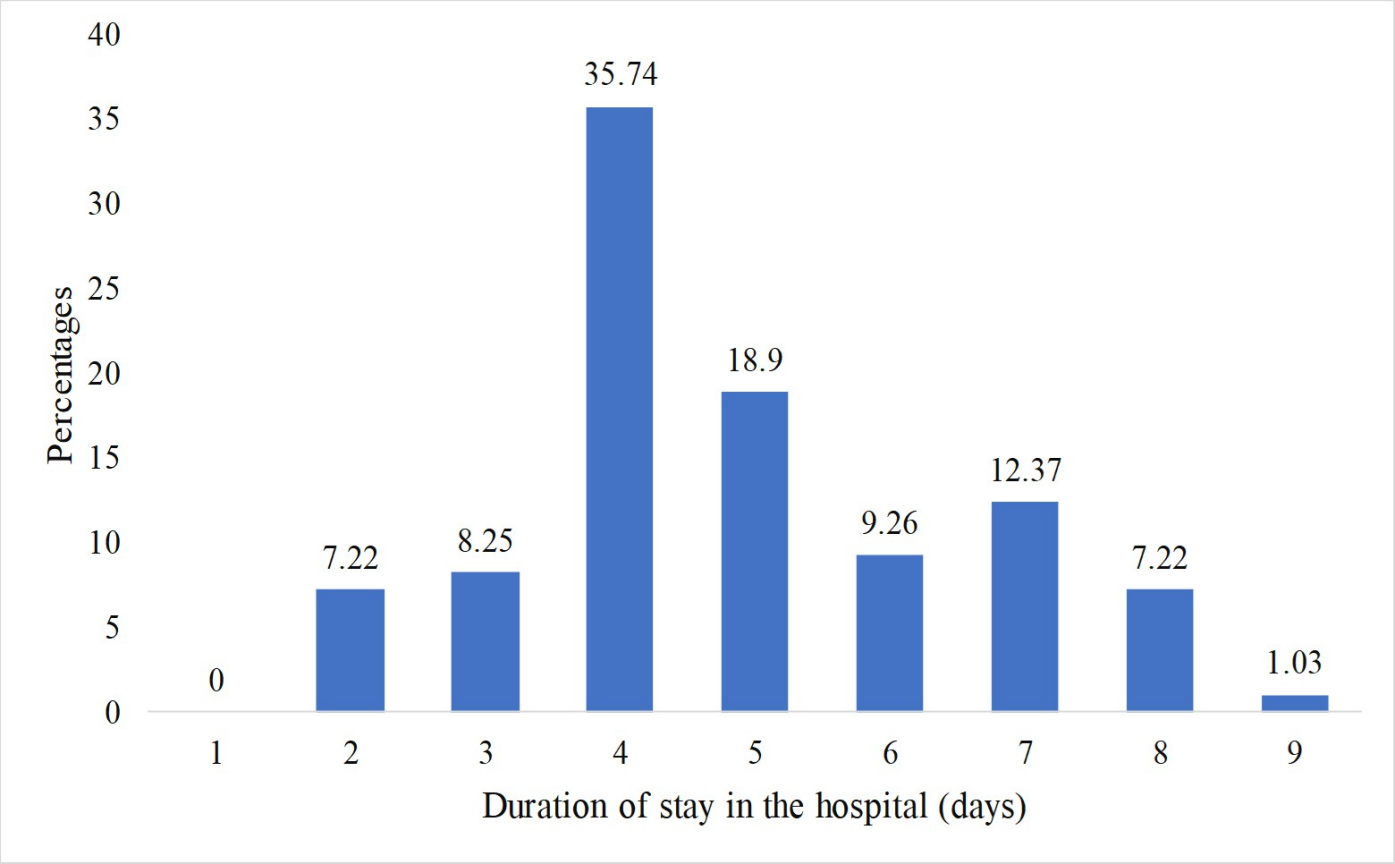
Distribution of the patients exhibiting varying recovery time after hospitalization.

Dengue patients had to stay in the hospital for an average of 4.9 days (SD = 1.652) with a range of 2 to 9 days before they recovered enough to be discharged from the hospital (Fig 6). Only 29.9% of patients stayed in the hospital for more than 5 days (Fig 6). Notably, a maximum of 35.74% of study patients got released on the 4^th^ day of admission into the hospital and no patient left the hospital on the day of hospitalization (Fig 6).

**Fig 7:**
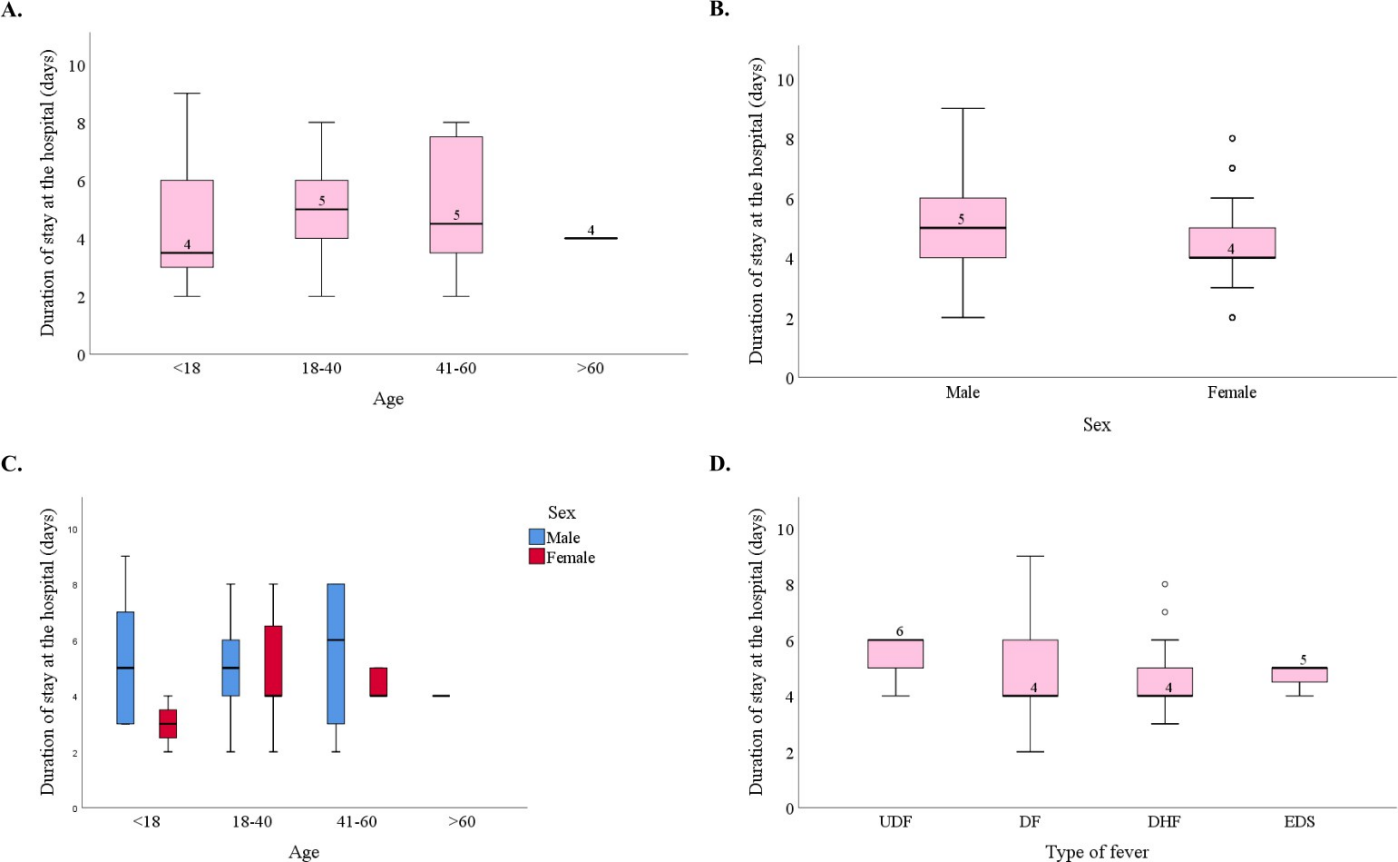
Duration of stay at the hospital (days) and its relationship with demographic factors and with the type of dengue fever observed in the patient. (A) Box plot showing the relation of age and duration of stay at the hospital. (B) Box plot showing the relation of gender and duration of stay at the hospital. (C) A clustered box plot showing interrelation among age, sex and duration of stay in the hospital. (D) Box plot showing the relation of type of dengue fever and duration of stay at the hospital. Circles (o) above the DHF box indicate outlier cases with extreme duration of stay at the hospital.

It was also evident from data that, patients from the age group of 41–60 years had to stay in the hospital for the highest number of days on average (5.1 days; 95%CI: 4.34–5.83) followed by patients of 18–40 and under 18 age groups who had an average hospital staying of 5 days (95%CI: 4.77–5.18) and 4.4 days (95%CI: 4.34–5.83) respectively (Fig 7A and S4 Table). Elder patients with more than 60 years of age stayed in the hospital for an average of 4 days (95%CI: 4.00–4.00) (Fig 7A and S4 Table). Male patients had to stay in the hospital for an average of 5 (95%CI: 4.81 to 5.27) days which is significantly longer than that of female patients who stayed in the hospital for an average of 4.5 (95%CI: 4.17 to 4.83) days (p < 0.01) (Fig 7B).

Difference between the median values of time of recovery between males and females was highest in patients who were less than 18 years of age as the median values were 6 and 3 respectively for males and females in this age group. The differences were 1 and 2 days respectively for age group 18–40 and 40–60 years. However, there was no female subject with more than 60 years of age (Fig 7C). No significant correlation was found at 0.05 level, between age groups and duration of stay in the hospital while considering “sex” as a control (No data shown).

No significant relationship was found between the type of fever and duration of stay in the hospital (p>0.05) (Fig 7D). However, maximum patients with UDF, DF, DHF and EDS stayed in the hospital for 6, 4, 4 and 5 days respectively before getting release permission from the hospital (Fig 7D).

### Linear model Output

In this longitudinal study, the independent variables (X) were measured at 6–9 time-points (days). The dependent variable Y denotes the number of days admitted to the hospital for dengue treatment. Thus, the analysis used repeated-measures independent variables and a dependent variable that was only measured once.

The analysis was performed in two steps: first, finding how the X variables varied with time across the individuals. Second, finding out how the estimates of the differential slopes and intercepts were associated with the duration of stay in the hospital. Thus, a conditional model (mixed-effects model) initially considered each X as the response. Using the standard two-level random-effects model with time in the fixed part and random slopes, we obtained the two parameters for each individual:

u_0j_, the precision-weighted estimate of the value of X as a differential from the mean X at the start across all individuals; and
u_1j_ - the precision-weighted estimate of the differential slope; positive values are those individuals who have increased their X more than the general trend, and negative values identify individuals who have increased their X at a lower rate than the general trend.

In the next step, standard regression models considered Y as the response, two covariates, Age and Sex of the patients, and four pairs of u_oj_ and u_1j_ as the predictors generated from the four independent variables (SBP, DBP, TMP, PLT). The step-by-step procedure gives the following model (Table 4).

**Table 4:** Linear model Output.

	as. numeric (DS)		
*Predictors*	*Estimates*	*CI*	*p*
(Intercept)	-68.23	-101.32–-35.14	**<0.001**
TMPr	2.10	0.33–3.88	**0.021**
TMPi	0.70	0.36–1.03	**<0.001**
SBPr	0.27	0.12–0.41	**<0.001**
DBPi	0.04	0.01–0.07	**0.021**
PLTr	0.05	0.03–0.06	**<0.001**
Observations	203		
R^2^/R^2^ adjusted	0.341/0.325		

It can be shown that the duration of stay at the hospital was positively associated with an increasing trend of temperature, systolic blood pressure and platelets count, and increasing differential of initial temperature and diastolic temperature from the respective overall means across all individuals. The study further reveals that the association between duration of stay and differentials in slopes for systolic blood pressure, platelets counts and differentials in intercepts for temperature were highly significant (p<0.01). In addition, the association of differentials in the slope for temperature and diastolic blood pressure with duration of stay in the hospital and were statistically significant (p<0.05). The fitted model explained 34.1% of the total variation in the hospital stay among the individual patients who participated in this study.

## Discussion

In this retrospective observational study, we aim to correlate the socio-demographic pattern, clinical parameters and consciousness of dengue patients of Bangladesh in 2019 to the occurrence, severity of the disease and clinical outcome. We also intend to track the temporal changes of various physiological parameters throughout the course of dengue treatment to construct a statistical model and define the variation in recovery time among individual study patients. It was noted that majority of the infected patients were male, urban resident, belonged to the age group of 18–40 years and required longer time to recover compared to females. Fever was the most common symptom observed in almost all the patients and a significant positive correlation was found between age and number of symptoms manifested in patients. Additionally, significant negative correlation were found for patients’ recovery time with both initial systolic and diastolic blood pressure as well as with the initial platelet count. Delayed hospitalization of the patients extended their duration of stay in the hospital while patients from 41–60 age group had to stay maximum number of days on average in the hospital. To the best of our knowledge this is the first report in Bangladesh on how the temporal trend of physiologic parameters (e.g., platelet count, blood pressure) and socio-demographic factors including age, gender, late hospitalization might have affected the clinical course of the disease and duration of recovery from Dengue in 2019 in Bangladesh. Findings obtained from this study may have clinical implications by exploiting them in the management of future dengue outbreak, in the hospitals for better patient care and in raising awareness among people as well.

In previous years dengue was considered predominantly as a childhood disease in most parts of the Southeast Asia [25]. The few sporadic dengue cases reported in Bangladesh before 2000 largely involved children aged 7–15 years [26–28] while the epidemiological pattern shifted more towards young adults (18–33 years) during the first dengue outbreak in early 2000s [29–31]. Current study showed that majority of the dengue patients were adults (18–40 years) which is in concordance with several recent investigations conducted in various South-Asian countries including Bangladesh, Singapore, Srilanka, India and other countries as well [18,20,29,32–36]. Lowered herd immunity of adults and decreased mortality rate in childhood infections might be responsible for this shift of dengue epidemiology towards older people [20]. It was observed that, after the sudden surge in 2000–2002, dengue transmission reduced during the period 2003 to 2016 [14] which may have lowered the herd immunity of the Bangladeshi adults against this infection in the following years. Interestingly, in recent years (from 2017) DENV3 and 4 are the most observed virus serotype while over 2003–2016, DENV1 and 2 strains were more prevalent [13,37]. Infection by a particular strain can only provide serotype specific life-long immunity and cross-immunity against other types is transient and may lead to complex clinical manifestations in case of secondary infection [37]. Hence, a lowered herd immunity in the young adults due to the reduced levels of dengue transmission over the past few years and lack of immunity against the recent virus serotype may be responsible for the massive dengue spreading among this age group during the 2019 outbreak in Bangladesh. In addition, presence of comorbidities [38], workplace transmission [33,39] and increased travel [40] among young adults may explain the reason of maximum prevalence of DENV infection in the age group of 18–40 years than children (<18 years) and older (41–60 and >60 years) population.

In this study, male patients outnumbered the female ones with a male-to-female ratio of 2.2: 1 which complies with many of the previous studies [12,18,20,32,41]. Anker and Arima demonstrated a consistent predominance of dengue cases in males compared to female via an epidemiological studies conducted in six Asian countries [41]. Such gender discrepancies can be explained in view of the social structure of the Asian countries. Males in South Asia are more likely to work outside than females, rendering them more vulnerable to the mosquito bites during day time either at their workplaces or while travelling. Additionally, male patients predominance in the access and utilization of the health care facilities are observed in Bangladesh. Reportedly, men attend or are taken to the health services more frequently than woman both in rural and urban areas and hence even if there was an equality in the number of incident dengue cases, this could have affected the reported figures [41–43]. Interestingly, in South America an opposite scenario was observed where the female dengue cases were either equal or higher than that of male [38,44]. However, it can not exclude the possibility of higher inherent susceptibility of males to DENV infection. The X chromosome contains several major miRNAs that play significant roles in different important immune regulations such as granulocytic differentiation, TLR mediated epithelial innate immune response, etc. but the Y chromosome lacks such miRNAs, thereby, leaving the males with certain immune disadvantages that may contribute to the higher infection rate in male population [45]. Moreover, male patients showed a significantly longer stay in the hospital than the female patients in this study. This finding might be explained by the X-linked microRNAs associated immune advantages in female as well [45].

Importantly, this study points out a major regional difference in the frequency of the disease occurrence. Most of the patients were found to be coming from urban area which conforms to the findings of several other recent studies [20,32]. Urban environment acts as better habitat for *Aaedes aegypti*. However, the fact that our concerned hospital was city-based and that the financial status, awareness and access to the health facilities of city dwellers are superior to that of rural people which may also contribute to this finding.

The current study showed that majority of the dengue patients suffered from DF followed by DHF which complies with the findings of other recent studies [46,47]. Hence, this study finds the predominant type of dengue during the 2019 outbreak as mild to moderate followed by a nominal number of severe and other forms of the disease. This study also indicated that almost all the dengue patients experienced fever as the most common clinical feature followed by other symptoms including bodyache, headache as well as various GI complications such as vomiting, anorexia and abdominal pain. However, we did not observe rash as a major symptom compared to the studies on early 2000s where it was found as one of the most predominent manifestations of dengue [48]. These findings on clinical manifestations are in agreement with several other studies conducted in recent years [18,32]. A significant positive correlation was noted between patient’s age and the number of symptoms exhibited which is in corroboration with the fact that DENV infected children mostly remain asymptomatic or display fewer symptoms [12].

It was evident from the analyzed data that both systolic and diastolic blood pressure were lower than normal blood pressure at the time of hospital admission which can be explained with the convention of plasma leakage due to increased capillary permeability in the critical phase of dengue which is 3 to 7 days after onset of fever [24]. Interestingly, most of the patients included in this study also became hospitalized during this phase [24]. Extended hospital days with compensatory oral fluid intake/IV therapy/transfusion are required to counter-measure this plasma leakage [4]. This fact explains the observed significant negative correlation of patients’ recovery time in this study with the initial lower SBP and DBP [4]. In addition, we found that, systolic blood pressure showed a more varying as well as decreasing tendency in hospitalized dengue patients compared to that of diastolic blood pressure. This explains the decrease in pulse pressure of dengue patients as SBP falls towards DBP also observed by Yeung W et al in 2020 [24]. However, the percentage of patients with deteriorating blood pressure reduced from day 1 to day 5 for both SBP and DBP and with the duration of stay, the pressures gradually went back to normal which indicates a positive outcome of treatment.

A major finding of this study was, a significantly negative correlation was found between the initial platelet count and the duration of stay of the patients in the hospital. This data indicates that the initial platelet count in dengue patients might act as a recovery parameter supporting the finding of Jayashree et al. in 2011 [23]. Present work also demonstrated that platelet count of dengue patients fell to nadir, on an average, on the third day of hospitalization and then gradually increased until day 8 or the last day of stay in the hospital indicating a positive outcome of treatment after admission into the hospital. This observation suggest to ensure extra close monitoring of patients when they are most likely to reach the lowest platelet count as prophylactic platelet transfusion therapy is not indicated for mild or moderate thrombocytopenic patients [49]. However, only 29.54% of the patients were found to be admitted into the hospital with mild/moderate thrombocytopenia and no patient with severe form of the parameter was recorded.

It did not escape from our observation that patient’s delay to get admitted into the hospital after the confirmation of dengue had a significantly positive relationship with their length of hospital staying. Similar correlation was observed between patients’ hospital stay and delay in hospitalization after the first onset of symptoms. These findings suggest that early awareness and clinical management can reduce the clinical complications and thereby, can reduce the hospital stay of patients by ensuring minimum deviation of blood pressure and platelet count. Patients from the rural area seek hospitalization quite late compared to the urban people and this difference was found to be significant in this study. Poor economic condition, lack of awareness and knowledge about dengue fever, transportation might play role behind this situation.

Average length of hospitalization of dengue patients and percentage of patients with more than 5 days of hospitalization were witnessed to be slightly higher in this study compared to some other recent studies conducted in neighbouring countries [25,50]. Lack of prompt hospitalization and/or sub-optimized management of dengue patients could be responsible for the situation. Patients of middle age had to stay longer in the hospital compared to the younger patients. Co-existence of multiple disorders and/or a relatively weaker immune systems compared to younger population might be the reason of this slow recovery in middle aged and older patients from dengue infection [51]. However, the least number of days of hospitalisation were showed by the elderly (>60 years) patients which could be due to a lack in the data collection in this investigation as not many patients were found in this group.

Finally, a statistical model was constructed that explained 34.1% of the total variation in the hospital stay among patients in terms of SBP, DBP, temperature and platelet count. Significant association of patients’ hospital stay with differentials in slopes for systolic and diastolic blood pressure and platelets counts suggests that lower trend in variation of these clinical features facilitate faster recovery of patients. This model might be helpful to better understand and predict the recovery speed of the patients based on their physiological parameters.

## Limitations and implications of the study

This study had certain limitations, for instances, it was conducted on a small sample of 336 in-patients of a tertiary level hospital, located in the capital city of Bangladesh, for a very short window of only two months. In addition, since the data were retrospectively collected from patients’ medical record, often we had to encounter random missing data which were not recorded appropriately in the patients’ medical record file by the responsible hospital authority. No data was found for patients with severe thrombocytopenia or any death cases. Hence, a prospective, multi-centered, larger sampled study needs to be conducted throughout the dengue season or, even for multiple years.

However, this study may have important clinical indications and implications in the management of dengue in Bangladesh as well as in other South Asian countries. Unlike the previously reported cases where rash was a common symptom of dengue, high temperature and GIT complications were the mostly observed symptoms in 2019 dengue outbreak suggesting there might be a shift in the symptoms of the disease. Therefore, this study recommends to conduct more strong mass awareness programs by the physicians and the Government on seeking clinical care as soon as people experience any of these symptoms. Another significant finding of this study is the considerable shift in the age of the dengue affected patients which warrants more awareness and extra vigilance of the young adults while staying outside home. This work also advices the male population to take necessary precautions against the disease specially during the peak dengue periods (July-October) because of their predominance of getting infected. Increased urbanization and travelling render dengue mostly a city endemic. Hence the Government and the policy makers of Bangladesh can take measures to expand the rural areas in a more planned way and minimize the effects of rapid urbanization. Also, decentralization of resources, facilities, manpower, academic and administrative institutions may help to reduce the tendency of travelling to a great extent which, in turn, may help to prevent the spread of dengue.

Patients with high fever, lower platelet count and reduced blood pressure on the day of admission required longer time to recover. Hence, physicians can monitor these patients closely and take extra care specially on the 3rd day of hospitalization. Although most of the recent dengue cases were mild to moderate, any delay in hospitalization after the onset of symptoms or diagnosis may extend the recovery time and lead to more complications. Therefore, knowledge about identification of the symptoms of dengue followed by immediate action are of utmost importance. This study may also aid the physicians to better understand the effects of different socio-demographic and clinical parameters of the patients on the clinical course of dengue and help ensuring early diagnosis, monitoring as well as a more optimized therapeutic protocol. Finally, the predictive statistical model depicting the significant correlations among various demographic/clinical parameters and patients’ recovery time may encourage the scientific communities to do further prospective, multi-centered, larger sampled studies and eventually construct other well-predictive statistical models for future dengue awareness, prevention and more optimized and comprehensive management of the disease in Bangladesh.

## Conclusion

Current study demonstrates the socio-demographic and clinical pattern of dengue fever during the 2019 dengue outbreak in Bangladesh and also presents the association of these parameters with the clinical course and treatment outcome of the disease on a day-to-day basis in a tertiary care hospital setting. The study demonstrates that initial high body temperature, lesser platelet counts and lower blood pressure lengthen the time to recover. Moreover, male and elderly patients’ predominance, a more urban nature of the disease and a shift in the common dengue symptoms were observed in the present work. Delay in the diagnosis and lack of seriousness about the disease also were responsible for the longer recovery time of the patients. Finally, the statistical model predicted that hospital stay could be lengthened with a rising trend of temperature, systolic blood pressure and presence of thrombocytopenia. Despite having some shortcomings including small sample size and some missing patients’ records, findings of this study may still prove to be beneficial for physicians to better understand the clinical course of the disease, for close monitoring of the patients with the above mentioned risk factors and to ensure improved patient management during future dengue outbreaks. This study also recommends conducting mass awareness programs by the Government and health care services to alert people about the potential socio-demographic risk factors, the common dengue symptoms, the changes in the disease pattern and to advice them to seek nearby medical help without delaying.

## Supporting information

S1 Fig Distribution of patients with different types of fever in specific age groups. (TIF)

S2 Fig Type of dengue fever versus Sex. (TIF)

S3 Fig Association of total number of symptoms in dengue patients with their age and/or gender. **(**A) Box plot depicting the pattern of total number of symptoms displayed by patients of various age groups (B) Mean differences for the total number of symptoms among various age groups. (C) A clustered box plot showing interrelation among age, sex and total number of symptoms displayed by a patient.
(TIF)

S4 Fig Occurance of different forms of thrombocytopenias in patients during their stay in the hospital (worst level observed were considered for each patient). (TIF)

S1 Table Distribution of age, sex and demography for the dengue patients (n = 336). (DOCX)

S2 Table Distribution of clinical features among the dengue patients studied in this study. (DOCX)

S3 Table Age and total number of symptoms displayed by a patient. (DOCX)

S4 Table Relation between the age range and average recovery time (duration of stay in the hospital). (DOCX)
