# Supplementary figures and images for: A retrospective study on the socio-demographic factors and clinical parameters of dengue disease and their effects on the clinical course and recovery of the patients in a tertiary care hospital of Bangladesh
Source: PLoS Negl Trop Dis. 2022 Apr 4;16(4):e0010297. doi: 10.1371/journal.pntd.0010297 (PMC8979461; doi:10.1371/journal.pntd.0010297)

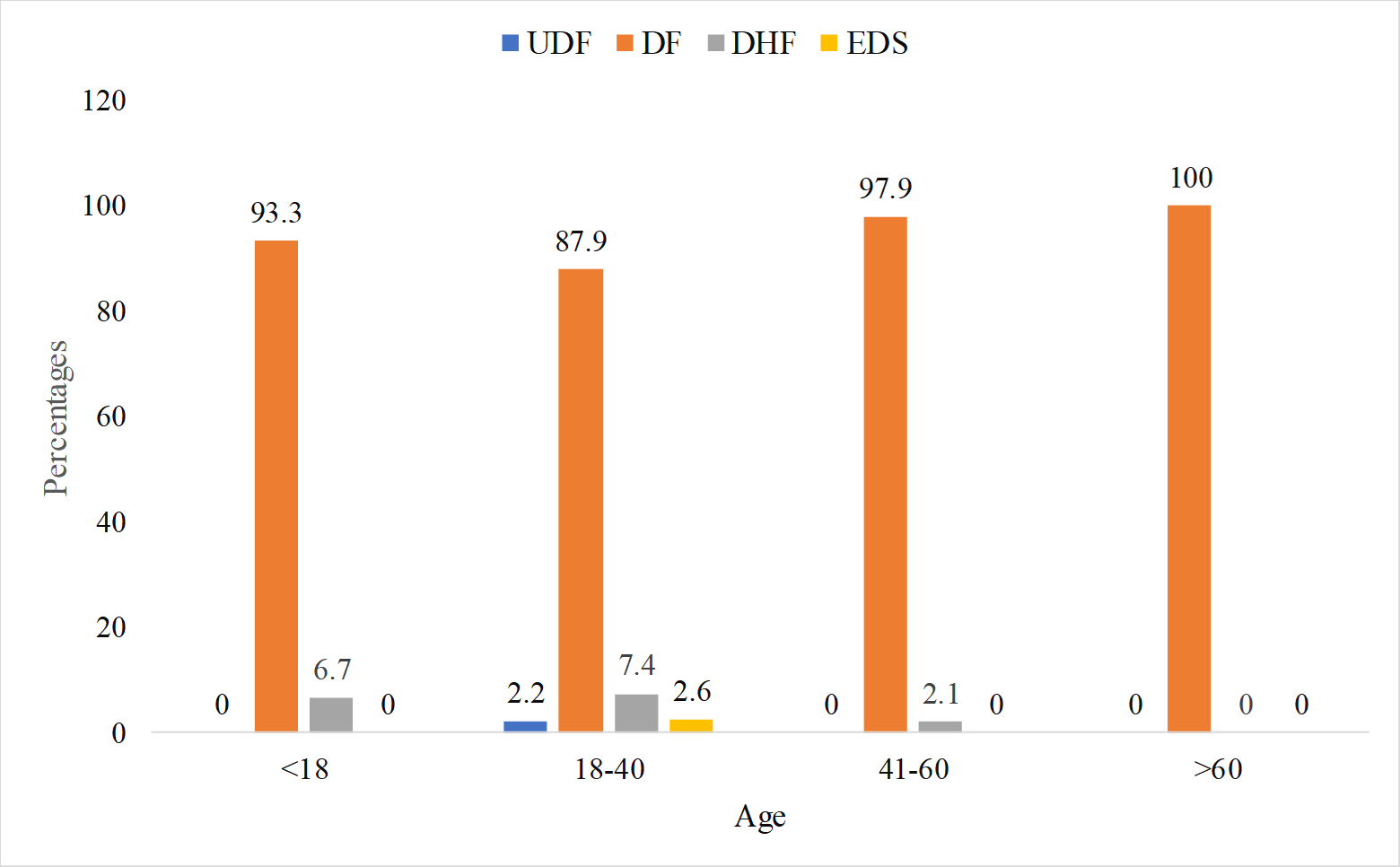

Supplement: S1 Fig — (TIF) [file pntd.0010297.s001.tif]

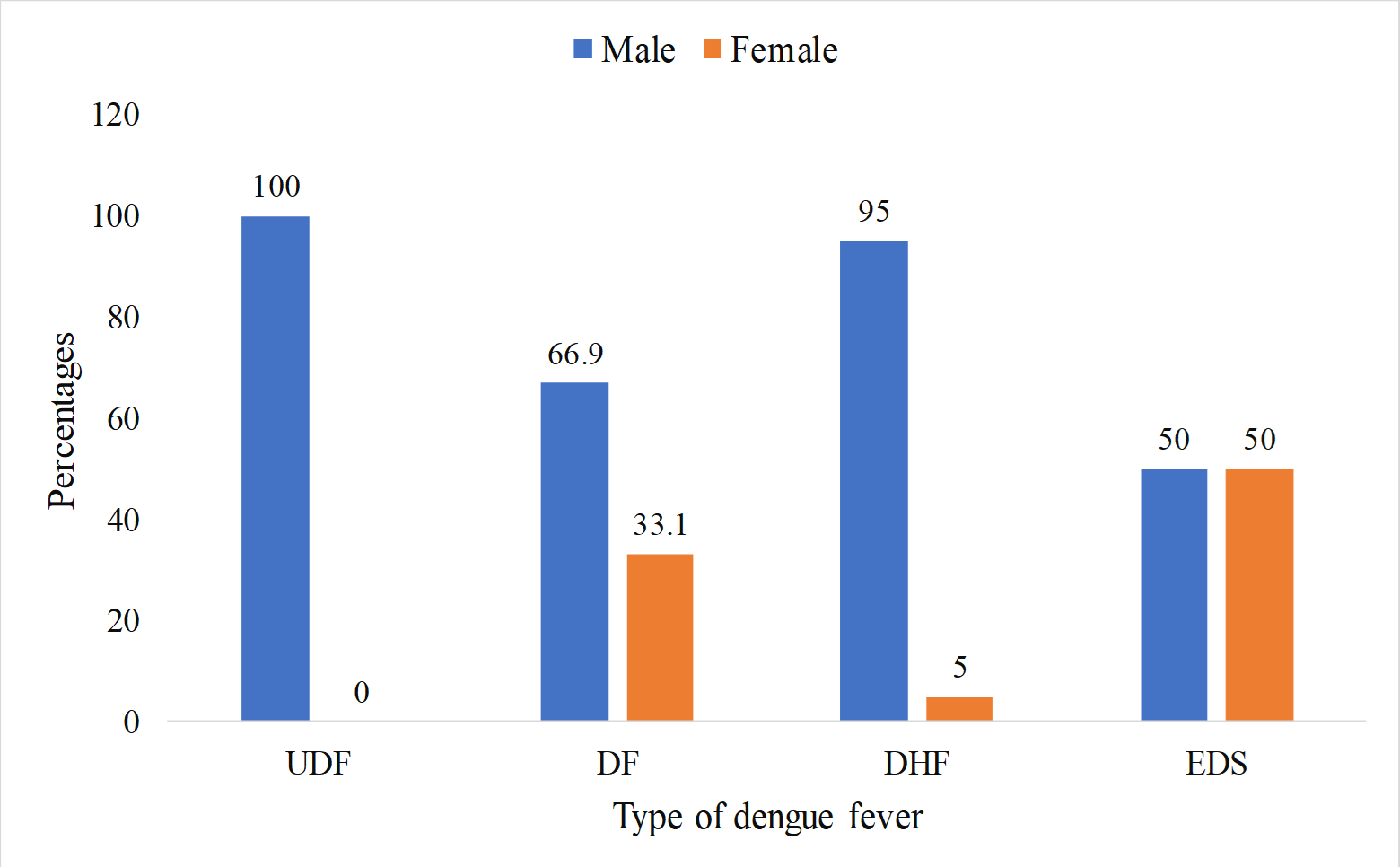

Supplement: S2 Fig — (TIF) [file pntd.0010297.s002.tif]

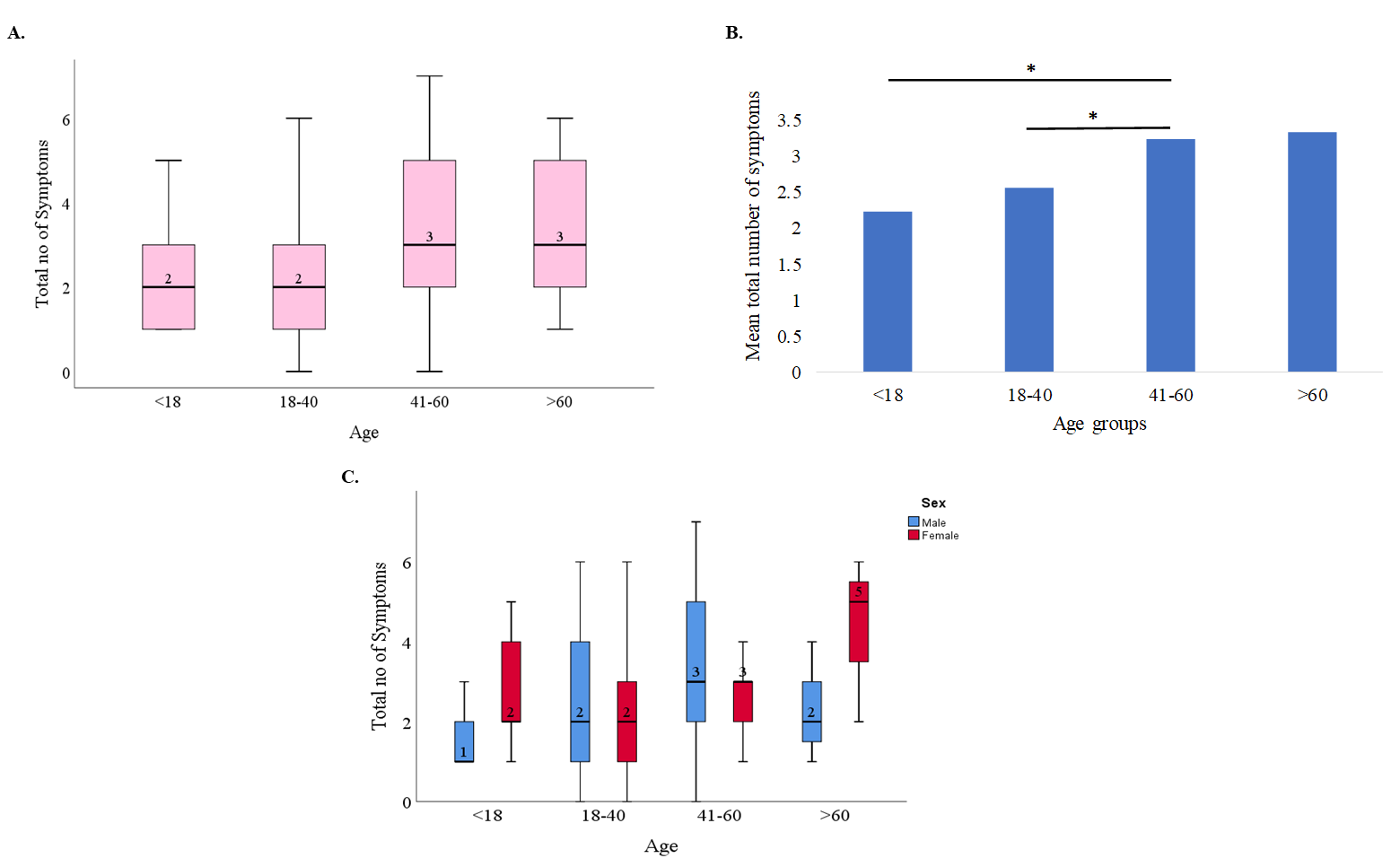

Supplement: S3 Fig — (A) Box plot depicting the pattern of total number of symptoms displayed by patients of various age groups (B) Mean differences for the total number of symptoms among various age groups. (C) A clustered box plot showing interrelation among age, sex and total number of symptoms displayed by a patient. (TIF) [file pntd.0010297.s003.tif]

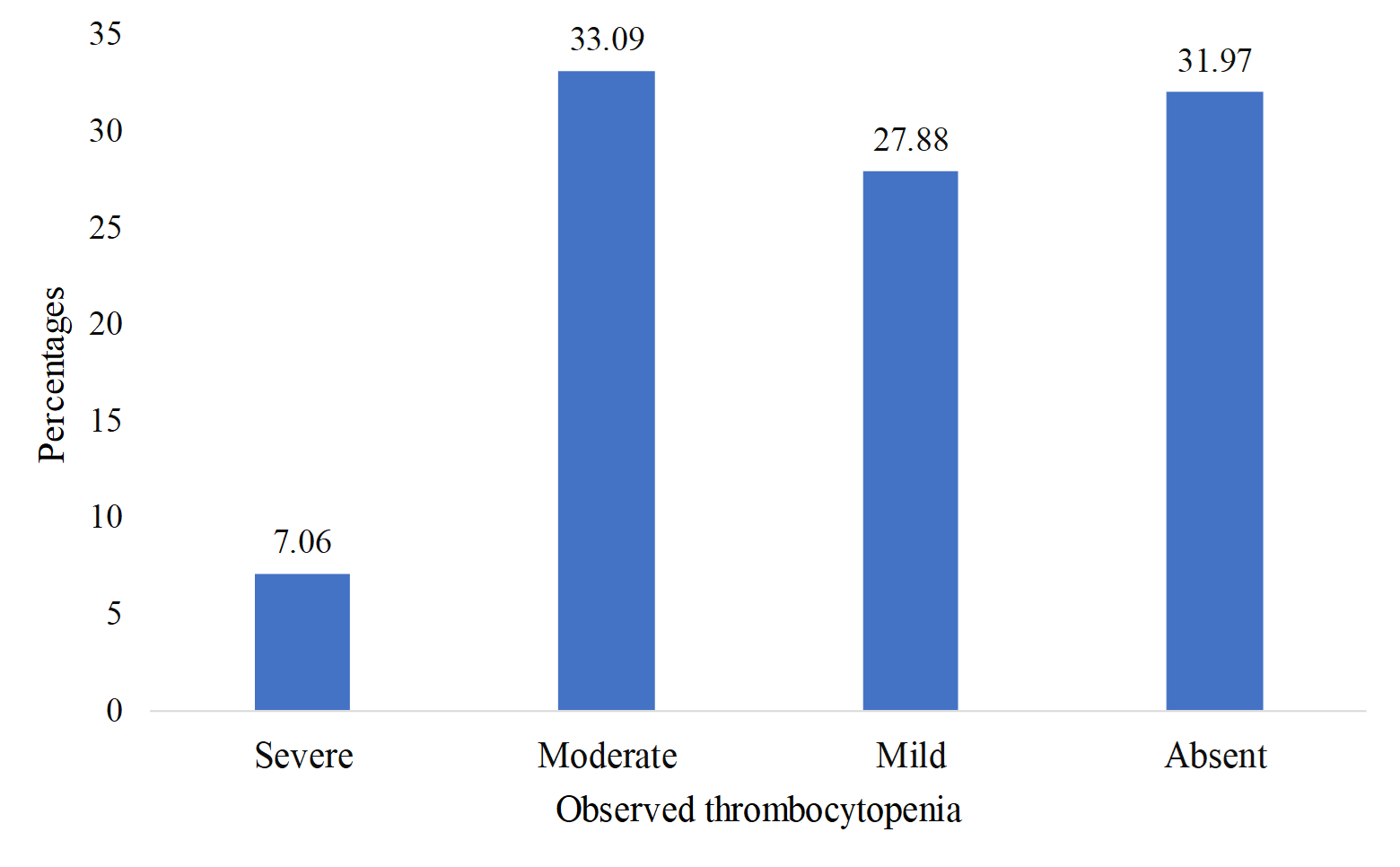

Supplement: S4 Fig — (TIF) [file pntd.0010297.s004.tif]
