# Supplementary material for: A retrospective study on the socio-demographic factors and clinical parameters of dengue disease and their effects on the clinical course and recovery of the patients in a tertiary care hospital of Bangladesh
Source: PLoS Negl Trop Dis. 2022 Apr 4;16(4):e0010297. doi: 10.1371/journal.pntd.0010297 (PMC8979461; doi:10.1371/journal.pntd.0010297)
Supplement: S1 Table — (DOCX) [file pntd.0010297.s005.docx]

**Table S1: Distribution of age, sex and demography for the dengue patients (n=336).**

|  | **No. of patients** | **Valid percentages (%)** | **Male*** | **Female*** |
| --- | --- | --- | --- | --- |
| **Age (years)** |  |  |  |  |
| <18 | 30 | 9.52 | 18 | 12 |
| 18-40 | 231 | 73.33 | 165 | 66 |
| 41-60 | 48 | 15.24 | 33 | 15 |
| >60 | 6 | 1.90 | 3 | 3 |
| **Sex** |  |  |  |  |
| Male | 231 | 68.75 | NA | NA |
| Female | 105 | 31.25 | NA | NA |
| **Demography** |  |  |  |  |
| Urban | 150 | 54.35 | NA | NA |
| Rural | 126 | 45.65 | NA | NA |

NA: not applicable. * Age data were available for 315 patients.
