# Supplementary material for: A retrospective study on the socio-demographic factors and clinical parameters of dengue disease and their effects on the clinical course and recovery of the patients in a tertiary care hospital of Bangladesh
Source: PLoS Negl Trop Dis. 2022 Apr 4;16(4):e0010297. doi: 10.1371/journal.pntd.0010297 (PMC8979461; doi:10.1371/journal.pntd.0010297)
Supplement: S2 Table — (DOCX) [file pntd.0010297.s006.docx]

**Table S2: Distribution of clinical features among the dengue patients studied in this study.**

| **Clinical manifestations** | **No. of study patients** | **Symptom observed (%)** |
| --- | --- | --- |
| Fever | 301^a^ | 98.0 |
| Body ache | 132 | 39.28 |
| Vomiting | 99 | 29.5 |
| Headache | 71 | 21.1 |
| Anorexia | 60 | 17.9 |
| Nausea (without vomiting) | 45 | 13.4 |
| Abdominal pain | 41 | 12.2 |
| Diarrhea | 26 | 7.7 |
| Rash | 18 | 5.4 |
| Malaise | 16 | 4.8 |
| Black stool | 13 | 3.9 |
| Cough | 12 | 3.6 |
| Backpain | 8 | 2.4 |
| Joint pain | 7 | 2.08 |
| Dehydration | 6 | 1.8 |
| Nasal bleeding | 6 | 1.8 |
| Respiratory distress | 5 | 1.5 |
| Slurry speech | 5 | 1.5 |
| Itchiness | 4 | 1.2 |
| Psychiatric problem | 4 | 1.2 |
| Edema | 3 | 0.9 |
| Chest pain | 3 | 0.9 |
| Gum bleeding | 3 | 0.9 |

^a^ 29 data were missing or not able to collect.
