# Supplementary material for: A retrospective study on the socio-demographic factors and clinical parameters of dengue disease and their effects on the clinical course and recovery of the patients in a tertiary care hospital of Bangladesh
Source: PLoS Negl Trop Dis. 2022 Apr 4;16(4):e0010297. doi: 10.1371/journal.pntd.0010297 (PMC8979461; doi:10.1371/journal.pntd.0010297)
Supplement: S3 Table — (DOCX) [file pntd.0010297.s007.docx]

**Table S3:** **Age and total number of symptoms displayed by a patient.**

| **Age group** | **<18** | **18-40** | **41-60** | **>60** |
| --- | --- | --- | --- | --- |
| Mean of total number of symptoms in a patient | 2.23^a^ | 2.56^b^ | 3.23^a,b^ | 3.33 |
| 95% Confidence interval (CI) | 1.78-2.69 | 2.37-2.75 | 2.73-3.73 | 1.27-5.40 |
| Std. error | 0.223 | 0.097 | 0.250 | 0.803 |

^a,b^ Age groups with similar superscript carry significant difference (at 0.05 level) in the mean number of symptoms between them.
