# Supplementary material for: A retrospective study on the socio-demographic factors and clinical parameters of dengue disease and their effects on the clinical course and recovery of the patients in a tertiary care hospital of Bangladesh
Source: PLoS Negl Trop Dis. 2022 Apr 4;16(4):e0010297. doi: 10.1371/journal.pntd.0010297 (PMC8979461; doi:10.1371/journal.pntd.0010297)
Supplement: S4 Table — (DOCX) [file pntd.0010297.s008.docx]

**Table S4:** **Relation between the age range and average recovery time (duration of stay in the hospital).**

| **Age group** | **<18** | **18-40** | **41-60** | **>60** |
| --- | --- | --- | --- | --- |
| Mean duration of stay in the hospital | 4.40 | 4.98 | 5.08 | 4.00 |
| 95% Confidence interval (CI) | 3.60-5.20 | 4.77-5.18 | 4.34-5.83 | 4.00-4.00 |
| Std. error | 0.391 | 0.106 | 0.368 | 0.00 |
